# Updated 2022 ACC/AHA Guideline Improves Concordance Between TTE and CT in Monitoring Marfan Snydrome and Related Disorders, but Relevant Measurement Differences Remain Frequent

**DOI:** 10.5334/gh.1322

**Published:** 2024-05-08

**Authors:** Johannes Kolck, Tobias Daniel Trippel, Karla Philipp, Petra Gehle, Dominik Geisel, Nick Lasse Beetz

**Affiliations:** 1 Charité – Universitätsmedizin Berlin, corporate member of Freie Universität Berlin and Humboldt-Universität zu Berlin, Department of Radiology, Augustenburger Platz 1, 13353 Berlin, Germany; 2 BIH (Berlin Institute of Health), Berlin, Germany; 3 Charité – Universitätsmedizin Berlin, corporate member of Freie Universität Berlin and Humboldt-Universität zu Berlin, Department of Internal Medicine –Cardiology, Charitéplatz 1, 10117 Berlin, Germany; 4 Deutsches Herzzentrum der Charité, Department of Cardiology, Angiology and Intensive Care Medicine, Augustenburger Platz 1, 13353 Berlin, Germany; 5 DZHK (German Center for Cardiovascular Research), partner site Berlin, Germany

**Keywords:** Marfan syndrome and related disorders, Monitoring aortic enlargement, Computed tomography, transthoracic echocardiography

## Abstract

**Background:**

Patients diagnosed with Marfan syndrome or a related syndrome require frequent aorta monitoring using imaging techniques like transthoracic echocardiography (TTE) and computed tomography (CT). Accurate aortic measurement is crucial, as even slight enlargement (>2 mm) often necessitates surgical intervention. The 2022 ACC/AHA guideline for Aortic Disease Diagnosis and Management includes updated imaging recommendations. We aimed to compare these with the 2010 guideline.

**Methods:**

This retrospective study involved 137 patients with Marfan syndrome or a related disorder, undergoing TTE and ECG-triggered CT. Aortic diameter measurements were taken based on the old 2010 guideline (TTE: inner edge to inner edge, CT: external diameter) and the new 2022 guideline (TTE: leading edge to leading edge, CT: internal diameter). Bland-Altman plots compared measurement differences.

**Results:**

Using the 2022 guideline significantly reduced differences outside the clinical agreement limit from 49% to 26% for the aortic sinus and from 41% to 29% for the ascending aorta. Mean differences were –0.30 mm for the aortic sinus and +1.12 mm for the ascending aorta using the 2022 guideline, compared to –2.66 mm and +1.21 mm using the 2010 guideline.

**Conclusion:**

This study demonstrates for the first time that the 2022 ACC/AHA guideline improves concordance between ECG-triggered CT and TTE measurements in Marfan syndrome patients, crucial for preventing life-threatening aortic complications. However, the frequency of differences >2 mm remains high.

**Clinical Relevance/Application:**

Accurate aortic diameter measurement is vital for patients at risk of fatal aortic complications. While the 2022 guideline enhances concordance between imaging modalities, frequent differences >2 mm persist, potentially impacting decisions on aortic repair. The risk of repeat radiation exposure from ECG-triggered CT, considered the ‘gold standard’, continues to be justified.

## Introduction

Marfan syndrome (MFS), with an estimated incidence of 2–3 per 10,000 individuals, is the most common genetic disorder affecting the aorta. Two less commonly inherited connective tissue disorders involving enlargement of the aorta are Loeys-Dietz syndrome (LDS) and the vascular form of Ehlers-Danlos syndrome (EDS) [1]. Untreated genetic aortic disorders, ranging between pathogenic variants in the FBN1, TGFBR1 or TGFBR2, SMAD2 and 3, as well as Col3A1 gene, lead to aortic dissection, rupture, and heart failure, shortening the life expectancy of affected individuals [2,3,4,5].

Cardiovascular monitoring is one of the key pillars in the diagnostic and therapeutic management of MFS and has greatly enhanced long-term survival of patients [6,7,8]. International guidelines recommend periodic imaging, using transthoracic echocardiography (TTE), computed tomography (CT) or magnetic resonance imaging (MRI), for the detection and management of changes in the aorta. CT and TTE are the most commonly used imaging modalities for aortic assessment. MRI, although preferred for younger patients, is less frequently employed due to its higher cost, longer examination times and limited availability [9]. To maintain consistency and accuracy of repeat aortic diameter measurement in patients with genetic aortic disease, it is advisable for them to undergo follow-up examinations at the same institution using a uniform imaging modality. This is particularly important when previous cross-sectional imaging studies have not provided adequate correlation due to differences in measurement conventions between institutions and imaging modalities [10]. Even though the latest guideline’s recommendations for aortic repair in MFS, vascular EDS and LDS differ, the accuracy of serial aortic diameter measurements is crucial in all settings for risk stratification and preoperative evaluation, as even slight progression of aortic enlargement may warrant surgical intervention for aortic repair [11,12].

As international guidelines recommend that aortic repair should be considered in patients with MFS and risk factors including size increase of ≥3 mm/year in serial examinations, measurement differences at the aortic root should not exceed 2 mm to ensure robust aortic monitoring [12,13]. In MFS patients, the limit is set at <±2 mm, aligning with recommendations indicating a 3 mm/year enlargement as the surgical intervention threshold. Exceeding a 2 mm difference is deemed unacceptable, impacting clinical decisions and potentially posing life-threatening risks, particularly in MFS cases.

The American College of Cardiology (ACC) and American Heart Association (AHA) have recently updated their measurement recommendations. The 2010 guideline recommended obtaining inner edge to inner edge measurement for TTE and external diameter measurement for CT [14,15]. In contrast, the updated 2022 ACC/AHA guideline now advocates leading edge to leading edge measurement in TTE and inner diameter measurements in CT imaging [12].

Considering the significant implications of aortic dimensions on treatment decisions, this study aimed to analyse the measurement variations between TTE and CT, while also assessing the level of agreement between measurements based on the updated 2022 ACC/AHA guideline compared to the previous 2010 guideline.

## Materials and Methods

### Study design

The study was approved by the institutional review board. Informed patient consent was waived by the ethics committee (Ethikkommission der Charité – Universitätsmedizin Berlin, registration number EA2/120/16). This single-centre retrospective study included 137 patients who were diagnosed with MFS or a related disorder. Each patient underwent both TTE and electrocardiography (ECG)-triggered CT for aortic diameter assessment. The study was conducted in accordance with the Declaration of Helsinki.

### Image acquisition and aortic diameter measurement

**Figure 1:**
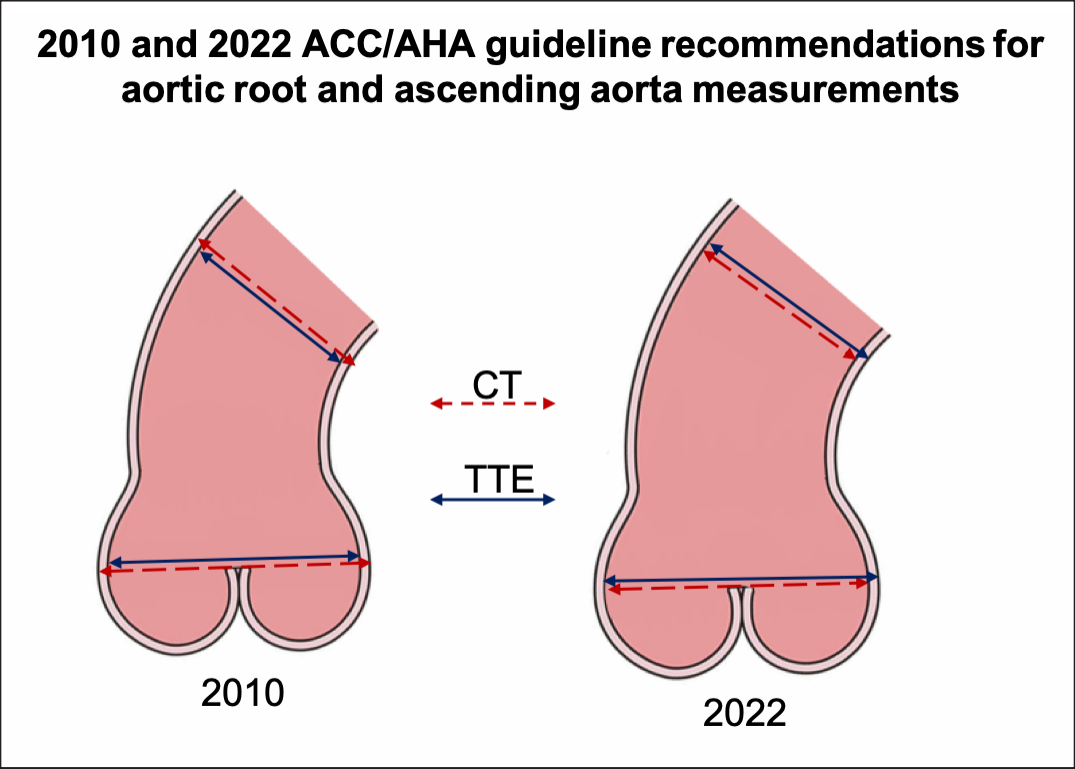
Schematic illustration depicting the varying measurement recommendations for CT and TTE at the aortic root and proximal ascending aorta. The American College of Cardiology (ACC) and American Heart Association (AHA) recently revised their measurement guidelines. The 2010 guideline suggested inner edge to inner edge measurement for TTE and external diameter measurement for CT [14,15]. However, the updated 2022 ACC/AHA guideline now recommends leading edge to leading edge measurement in TTE and inner diameter measurements in CT imaging [12] (adapted from Isselbacher, E.M., et al. [12]).

All patients referred to the Department of Radiology underwent imaging using a single-source 256-row CT scanner (Revolution CT, General Electric, Milwaukee, USA). An iodinated contrast medium was injected intravenously to enhance image quality. A two-step scanning strategy was employed, starting with an axial ECG-triggered whole-heart scan including the aortic root during diastole. This scan was followed by a helical scan of the entire aorta. Bolus tracking with SmartPrep (General Electric, Milwaukee, USA) was utilised to ensure adequate opacification of the aorta. Aortic diameters were measured using double-oblique multiplanar reconstructions perpendicular to the course of the aorta (Figure 1). Measurements were taken at the levels of the aortic annulus, aortic sinus, sinotubular junction and the largest diameter of the ascending aorta (between the aortic arch and sinotubular junction) using Visage software (Visage Imaging, Berlin, Germany) according to both the 2010 and 2022 ACC/AGA guidelines, measuring the external and internal diameter, respectively. At the level of the aortic sinus, the largest non-anatomically landmarked diameter at the mid-sinus level was recorded. All measurements were performed by a highly experienced radiologist with over seven years of expertise in cardiovascular imaging. For comparison with TTE, only the two largest diameters at the aortic root (aortic sinus and ascending aorta) were utilised.

In addition to CT imaging, all patients underwent two-dimensional TTE using the Epic 700 cardiac ultrasound machine (Philips, Amsterdam, the Netherlands) at both initial work-up and during follow-up. Aortic diameters at the aortic sinus and the ascending aorta were measured during diastole, employing the inner edge to inner edge and leading edge to leading edge measurement approaches. Following the 2010 guideline for echocardiography, the internal diameter was measured perpendicular to the axis of blood flow and at the aortic root, the widest diameter, typically at the mid-sinus level, was used. Following the 2022 guideline for echocardiography, the aortic root diameter was evaluated in both short- and long-axis images, measuring the maximum sinus-to-sinus diameters (L to R, N to R, and N to L) through multiple assessments, with the maximum value being recorded. These examinations and measurements were performed by a cardiologist and a cardiac surgeon, both board-certified with over 20 years of experience in echocardiography. The procedures adhered to an established and standardized protocol based on current echocardiography guidelines. TTE and CT measurements were conducted by separate readers. The CT measurement reader was blinded to the results of TTE measurements, and the two readers for the TTE measurements were also blinded to the CT measurements. Each imaging modality was measured once. Patients were included only if the time interval between the two imaging studies was less than six weeks.

### Statistical analysis

Means and standard deviations were calculated for diameters measured with both TEE and CT. To assess the disparities between the two methods of aortic diameter measurement, Bland-Altman plots were utilised. This approach was chosen as it is more adept at revealing pertinent differences compared to simply comparing means or conducting correlation analyses [16]. In Bland-Altman plots, the lower and upper tolerance range indicates acceptable limits for differences between two measurement methods. Typically, the mean difference is calculated, and the 95% limits of agreement are established by adding and subtracting 1.96 times the standard deviation. However, in contrast to this conventional approach, the acceptable clinical limit of agreement in Bland-Altman analysis is a pre-established threshold, often determined based on clinical considerations [16,17]. The predefined acceptable clinical limit of agreement was set at <+/–2 mm for the difference observed between the two measurement techniques. For each of the two guidelines and each of the two measurement sites (aortic sinus and ascending aorta), relative and absolute measurement differences outside the allowable tolerance, mean, standard deviation, lower tolerance range, and upper tolerance range were calculated. The data analyses were performed using IBM SPSS Statistics version 27 (IBM, Armonk, New York, USA).

## Results

### Study population

Our study included a total of 137 participants, among them 85 males and 52 females. Mean age at the time of TTE was 35.57 years. There were 103 patients (75.2%) diagnosed with Marfan syndrome, while 10 (7.3%) had Ehlers-Danlos syndrome and 14 (10.2%) had Leuys-Dietz syndrome. Ten study participants (7.3%) had other conditions associated with aortic enlargement. None of the individuals included in the study exhibited a bicuspid aortic valve. Drug treatment consisted of renin-angiotensin-aldosterone system (RAAS) inhibitors in 107 (78.1%) patients, beta-blocker medication in 25 (18.2%), and combined intake of both drugs in 15 patients.

### Aortic measurement

For each patient, aortic diameters were determined by both CT and TTE according to the 2010 and 2022 ACC/AHA guidelines. Mean aortic sinus diameters measured from CT were 43.2 mm and 42.0 mm using the external edge and internal edge method, respectively. Mean CT diameters of the ascending aorta were 36.2 mm and 35.1 mm, respectively. Standard deviations for these measurements ranged from 5.8 to 7.7 mm. In addition, the mean aortic anulus diameters measured from CT were 33.2 mm and 32.2 mm using the external edge and internal edge method, respectively. Mean CT diameters of the sinotubular junction were 31.6 mm and 30.7 mm, respectively. Standard deviations for these measurements ranged from 5.2 to 6.9 mm. Mean diameters measured from TTE were 40.5 mm for the aortic sinus and 35.0 mm for the ascending aorta using the inner edge method according to the 2010 ACC/AHA. Under the 2022 guidelines, mean diameters using leading edge to leading edge measurement were 41.7 mm for the aortic sinus and 36.2 mm for the ascending aorta. Standard deviations for these measurements ranged from 5.6 to 7.5 mm. The results obtained are summarised in Tables 1 and 2.

**Table 1:** Mean aortic diameters based on CT.


	CT			

	EXTERNAL DIAMETER, 2010 GUIDELINE		INTERNAL DIAMETER, 2022 GUIDELINE	

**Site**	**Aortic sinus**	**Ascending aorta**	**Aortic sinus**	**Ascending aorta**

**Mean and SD (mm)**	**43.2** ± **5.8**	**36.2** ± **7.7**	**42.0** ± **5.8**	**35.1** ± **7.6**

	**EXTERNAL DIAMETER, 2010 GUIDELINE**		**INTERNAL DIAMETER, 2022 GUIDELINE**	

**Site**	**Aortic annulus**	**Sinotubular junction**	**Aortic annulus**	**Sinotubular junction**

**Mean and SD (mm)**	**33.2** ± **5.2**	**31.6** ± **6.7**	**32.2** ± **5.2**	**30.7** ± **6.9**


**Table 2:** Mean aortic diameters based on TTE.


	TTE			

	INNER EDGE, 2010 GUIDELINE		LEADING EDGE, 2022 GUIDELINE	

**Site**	**Sinus**	**Ascending aorta**	**Sinus**	**Ascending aorta**

**Mean and SD (mm)**	**40.5** ± **5.6**	**35.0** ± **7.4**	**41.7** ± **5.5**	**36.2** ± **7.5**


### Evaluation of divergence between the two imaging modalities

**Figure 2:**
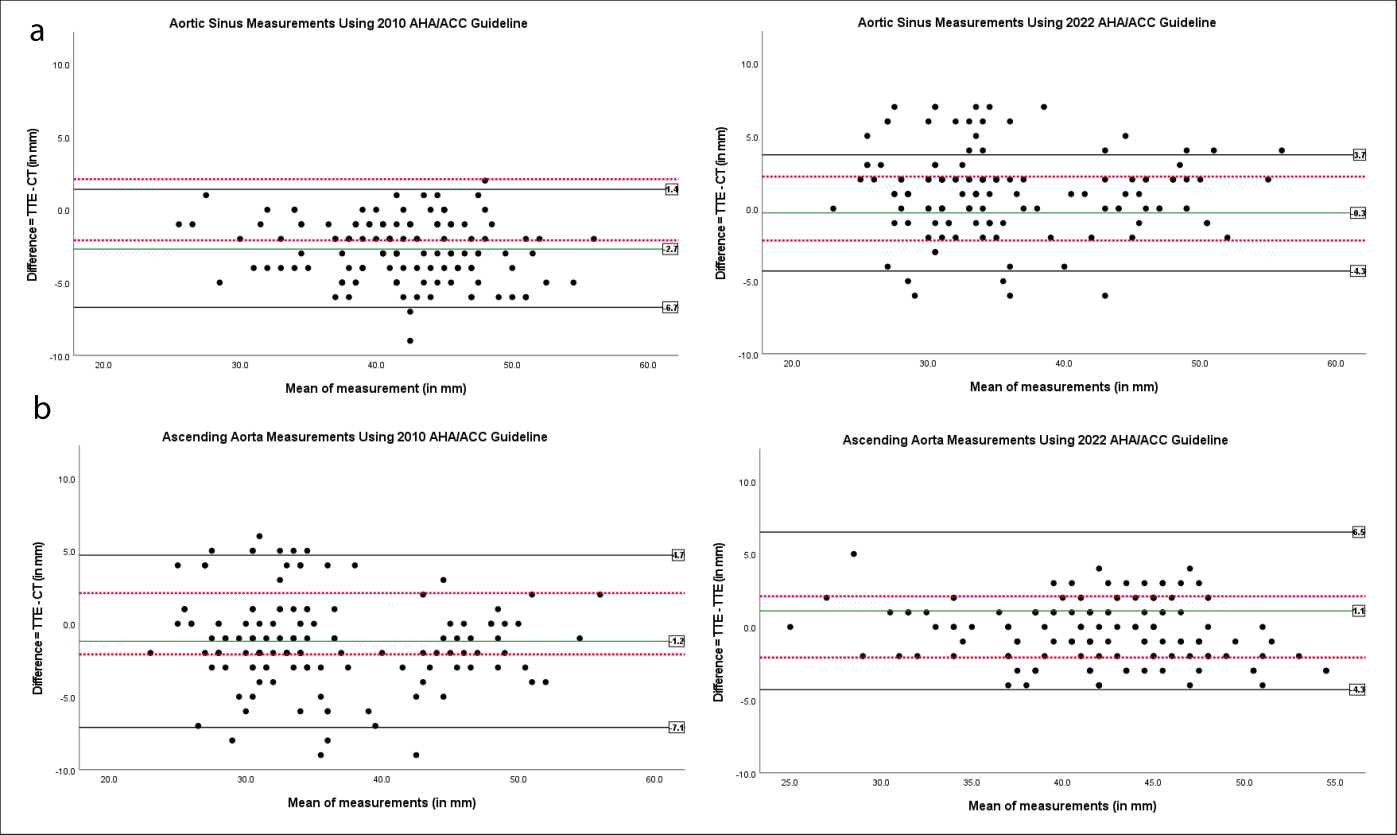
Bland-Altman plots depicting differences in diameter measurement at the aortic sinus **(a)** and at the level of the ascending aorta **(b)** applying both the 2010 AHA/ACC guideline and the 2022 AHA/ACC guideline. Dotted red line: clinically acceptable limit of agreement for difference (+/–2 mm). Green line: agreement bias. The frequency of measurement differences outside the acceptable clinical agreement limit of difference (outside the range of the lower and upper dotted red line) was significantly lower using the 2022 AHA/ACC guideline.

All 137 patients underwent CT and TTE examinations of the aorta, in accordance with the 2022 and 2010 ACC/AHA guidelines. Measurements were obtained at the aortic sinus and at the level of the ascending aorta. To estimate measurement deviations, the results were visualized as Bland-Altman plots (Figure 2a–b). The predefined acceptable clinical limit of agreement was set at <+/-2 mm for the difference observed between the two measurement techniques advocated in the 2010 and 2022 guidelines. When the 2022 method was used for diameter measurement, differences exceeding the acceptable tolerance range were observed in 35 cases (26%) for the sinus site and 40 cases (29%) for the ascending aorta site. In comparison, for the 2010 guideline, the number of cases exceeding the acceptable tolerance range was 67 (49%) for the sinus site and 56 (41%) for the ascending aorta site. The mean deviation from the reference value for the 2022 guidelines was –0.30 mm for the sinus site and 1.12 mm for the ascending aorta site. Conversely, under the 2010 guidelines, the mean deviation was –2.66 mm for the sinus site and –1.21 mm for the ascending aorta site. Standard deviations for these measurements ranged from 2.05 to 3.01 mm. Considering a tolerance factor of 1.96, we calculated a lower tolerance range for the 2022 guideline of –4.3 mm for the sinus site and –4.3 mm for the ascending aorta site, while the upper tolerance range was 3.7 mm and 6.5 mm, respectively. Similarly, for the 2010 guideline, the lower tolerance range was –6.7 mm for the sinus site and –7.1 mm for the ascending aorta site, with an upper tolerance range of 1.4 mm for the sinus site and 4.7 mm for the ascending aorta site. These results are compiled in Table 3.

**Table 3:** Overview of the measurement differences between CT and TTE in the assessment of the aortic diameter, depending on the application of the current 2022 and the older ACC/AHA guideline from 2010. The agreement bias represents the average difference between the two applied modalities. The lower and upper tolerance range are calculated by adding and subtracting 1.96 times the standard deviation the agreement bias.


	MEASUREMENT DIFFERENCES BETWEEN CT AND TTE			

GUIDELINE VERSION	2010		2022	
	
SITE	SINUS	ASCENDING AORTA	SINUS	ASCENDING AORTA

**Measurement differences outside acceptable tolerance (n, %)**	**67 (49%)**	**56 (41%)**	**35 (26%)**	**40 (29%)**

**Agreement bias (mm)**	**–2.66**	**–1.21**	**–0.30**	**1.12**

**Standard deviation of bias (mm)**	**2.06**	**3.01**	**2.05**	**2.77**

**Factor of 1.96**	**1.96**	**1.96**	**1.96**	**1.96**

**Lower tolerance range**	**–6.7**	**–7.1**	**–4.3**	**–4.3**

**Upper tolerance range**	**1.4**	**4.7**	**3.7**	**6.5**

**Total (n)**	**137**	**137**	**137**	**137**


## Discussion

The present study investigated the extent of agreement between aortic diameters measured by ECG-triggered CT and TTE in light of the recently updated ACC/AHA guideline. While discrepancies between mean values obtained with the two measurement methods appear manageable, on an individual level, Bland-Altman plots reveal a relevant measurement bias in aortic diameter measurement using CT and TTE. Mean differences in measurement were -0.30 mm for the aortic sinus and +1.12 mm for the ascending aorta using the method advocated in the new 2022 guideline, compared to –2.66 mm for the aortic sinus and +1.21 mm for the ascending aorta using the old 2010 guideline. Measurement differences outside the acceptable tolerance, defined as deviations >+/-2 millimeters, were substantial with 49% of cases at the sinus and 41% at the level of the ascending aorta a following the 2010 ACC/AHA guideline. Use of the new guideline significantly improved concordance between ECG-triggered CT and TTE measurements with deviations beyond the acceptable range dropping to 26% for the sinus and 29% for the ascending aorta. Significantly, this study employs Bland-Altman plots rather than relying on correlation analysis or a simple comparison of mean differences between two measurement methods. This approach enables the identification of substantial individual measurement differences in the context of this potentially life-threatening disease.

Enlargement of the aortic root is a crucial feature in the diagnosis of genetic aortic diseases and is strongly linked to a heightened risk of aortic dissection, consequently leading to increased mortality of affected individuals [2,18,19]. Measurement of aortic diameters in patients with MFS or associated disorders is of paramount importance in clinical practice to predict the risk of aortic complications and thus ensure timely initiation of aortic repair [8,20,21,22,23]. Generally, surgery is advised for MFS patients when aortic diameter is 50 mm or greater and considered for those with additional risk factors at diameters ≥45 mm. In patients with LDS and aortic dilatation, genetic variants, aortic diameter, growth rate, extra-aortic characteristics, family history, patient age and gender should be considered, in addition to patient and physician preferences when deciding on prophylactic replacement of the aortic root and ascending aorta. In vascular Ehlers-Danlos syndrome, surgical repair carries higher risks due to vessel fragility and associated bleeding complications. Rapid growth of the arterial aneurysm or dissection warrants treatment, but there is no data on thresholds for the diameter of prophylactic surgical intervention. As a result, decisions about aortic and vascular branch aneurysms and dissections are made by a multidisciplinary aortic team and decided jointly [11,12]. Most commonly, aortic diameter measurements are performed using either ECG-triggered CT or TTE. Since even minor changes in diameter may indicate surgery, the precision and consistency of aortic diameter measurement is of utmost relevance [22,23,24,25]. The recently updated 2022 ACC/AHA guideline advocates leading edge to leading edge measurement in TTE and inner diameter measurement in CT, replacing the earlier recommendation to obtain inner edge to inner edge measurements for TTE and outside diameters for CT [14]. The novel findings we present here demonstrate the potential of the updated imaging recommendations stated in the 2022 ACC/AHA guideline to enhance the agreement between ECG-triggered CT and TTE aortic diameter measurements in patients diagnosed with MFS or a related disorder. This is particularly relevant given that even slight progression of aortic enlargement (>3 mm) often necessitates surgical intervention [13].

Despite the notably improved agreement between the two modalities, the number of deviations outside the tolerance range remains substantial, at almost 30%. The problem of modality-dependent measurement differences has been intensively discussed in the past, with various authors working towards improving measurement consistency, sometimes proposing conflicting approaches. Notably, in 2015, Asallem and their team reported an enhancement in measurement agreement using the ‘inner-to-inner’ approach. However, just a year later, Rodriguez-Palomares and colleagues advocated for the ‘leading edge to leading edge’ measurement technique, which is now part of the 2022 guideline [26,27]. While several authores highlighted underestimation of aortic diameter in TTE compared to CT [28,29], admirably high concordance was observed between CT and MRI [30]. Our findings confirm these observations, demonstrating consistently lower mean aortic diameters in TTE, particularly in the sinus region. Beyond differences in measurement accuracy, each of the two modalities have their upsides and downsides. TTE is widely available, comes at a low cost, and allows additional assessment of cardiac functionality, such as mitral valve prolapse and aortic regurgitation, which are frequent in MFS [31]. The most relevant disadvantage of CT is exposure to ionizing radiation. Even though relevant dose reduction can already be achieved when state-of-the-art CT scanners and adjusted CT protocols are used, exposure is a concern in young patients who require repeat imaging and are thus exposed to potentially harmful amounts of ionizing radiation [32,33]. In general, CT is considered the preferred method for measuring aortic diameter in preoperative or preinterventional planning. This preference is based on its wide availability, high accuracy, and superior performance for detection of aortic dissection compared with both TTE and MRI [34,35,36].

In our study the frequency of measurement differences outside the acceptable clinical agreement limit of difference was significantly lower using the 2022 AHA/ACC guideline. However, the frequency remained relatively high which might be attributed by three main reasons. First, the often-overestimated TTE measurements cannot sufficiently adjust to thoracic deformity and/or an elongated ascending aorta typical in MFS, whereas aortic diameters taken from CT are determined from double oblique multiplanar reconstructions perpendicular to the course of the aorta. Second, an exatic proximal coronary artery is a common phenomenon in patients with MFS and might falsely lead to false overestimation by TTE [37]. Third, CT analysis have revealed that the aortic root is normally asymmetric and these asymmetries may contribute to discrepancies between different measurement methods [38].

Although the study provides valuable insight into the comparison of imaging recommendations for measuring aortic size in patients with MFS and related disorders, there are some limitations to be considered: Firstly, the retrospective design may introduce a selection bias and hinder robust results. The relatively small sample size may not fully represent the diversity of the patient population. Differences in imaging, demographics, and disease severity between different medical institutions may not be accounted for in this single centre analysis. Measurement accuracy depends on imaging precision and operator skill, potential human error may affect results. While the study focuses on differences in imaging measurements, it does not directly assess their impact on clinical outcomes or disease progression.

In conclusion, this study provides evidence that the updated imaging recommendations of the 2022 ACC/AHA guideline can improve the concordance between ECG-triggered CT and TTE in measuring aortic diameter in patients with Marfan syndrome and related disorders. However, despite the improvements, measurement differences beyond the acceptable clinical agreement limit remain a concern. These findings underscore the ongoing need for accurate and standardized imaging techniques to guide clinical decision-making in this high-risk patient population.

## Data Accessibility Statement

All data generated or analysed during the study are included in this published paper.
